# Therapeutic Implications of the Drug Resistance Conferred by Extracellular Vesicles Derived from Triple-Negative Breast Cancer Cells

**DOI:** 10.3390/ijms24043704

**Published:** 2023-02-12

**Authors:** Yong Weon Yi

**Affiliations:** Department of Biochemistry, College of Medicine, Dankook University, Cheonan-si 31116, Chungcheongnam-do, Republic of Korea; yongweon_yi@dankook.ac.kr; Tel.: +82-41-550-3974

**Keywords:** anticancer therapeutics, drug resistance, extracellular vesicles, exosomes, triple-negative breast cancer, tumor cell-derived extracellular vesicles

## Abstract

Anticancer drug resistance is a significant impediment in current cancer treatment. Extracellular vesicles (EVs) derived from cancer cells were recently acknowledged as a critical mechanism of drug resistance, tumor progression, and metastasis. EVs are enveloped vesicles comprising a lipid bilayer that transfers various cargo, including proteins, nucleic acids, lipids, and metabolites, from an originating cell to a recipient cell. Investigating the mechanisms whereby EVs confer drug resistance is still in the early stages. In this review, I analyze the roles of EVs derived from triple-negative breast cancer cells (TNBC-EVs) in anticancer drug resistance and discuss strategies to overcome TNBC-EV-mediated drug resistance.

## 1. Introduction

Recent advances in anticancer drugs have greatly improved cancer patient survival [1,2]. For example, 1 year and 5 year net survival increased between 1995 and 2014, in 3,764,543 eligible cancer cases, and from 19 jurisdictions spanning most cancer types [1]. Survival rate increases are evident in high-income countries. The 5 year mortality rate in adolescents and young adults with primary cancers decreased from 6.8% in those diagnosed between 1975 and 1984 to 4.2% between 2005 and 2011 [2]. These improvements were possible primarily through the development of targeted therapeutics. For example, seventy-one small-molecule protein kinase inhibitors (PKIs) were approved by the US Food and Drug Administration (FDA) as of 29 May 2022 [3]. Over the last thirty-five years, the US FDA has approved 100 monoclonal antibodies for treating various diseases, including cancers [4]. Novel therapeutic modalities, such as chimeric antigen receptor (CAR)-engineered cell therapies and antibody–drug conjugates (ADCs), have also contributed to the overall success of anticancer therapeutics [5,6,7,8,9].

The emergence of resistance, however, impedes the efficacy of these anticancer therapeutics, and represents another challenge to successful cancer treatment [10]. Two types of drug resistance are intrinsic (de novo or primary), resistance that exists before the use of a drug, and acquired (or secondary), resistance that develops during drug treatment [8,11]. Drug resistance occurs via many mechanisms, including increases in drug efflux, mutations in oncogenes and/or tumor suppressor genes, compensatory survival pathway activations, and DNA damage repair [10,12].

Triple-negative breast cancer (TNBC) is aggressive and accounts for up to 20% of breast cancer types [13,14,15,16,17]. TNBC is characterized by a lack of the expression of hormone receptors, both estrogen (ER) and progesterone (PR) receptors, and no amplification of human epidermal growth factor receptor 2 (HER2), leading to limited application in targeted therapy [11,13,14,18]. Specifically, TNBC has an intrinsic resistance against therapeutics targeting tyrosine kinase receptors, such as epidermal growth factor receptor (EGFR) and MET, despite their frequent amplification [11,13,18,19,20,21,22,23,24,25,26]. Understanding the mechanism of drug resistance in TNBC may reveal alternative treatment options that overcome TNBC’s refractiveness to targeted therapeutics.

Mounting evidence suggests that the secretome, including cytokines, growth factors, extracellular vesicles (EVs), and circulating nucleic acids, plays a vital role in drug resistance [27,28,29,30,31,32]. EVs, from cancer cells or stromal cells in the tumor microenvironment (TME), are key mediators of drug resistance. EVs are membranous vesicles derived from living cells and are categorized, according to their biogenesis, as exosomes (30–200 nm in diameter), microvesicles (also known as ectosomes, 100–1000 nm), and apoptotic bodies (500–2000 nm) [33,34,35]. EVs cause phenotypic changes in recipient cells by conveying various cargo, including proteins, lipids, nucleic acids, and metabolites, from parental cells to recipient cells [33,34,35,36]. EVs also contribute to cell-to-cell communication, a critical component of tumorigenesis and metastasis [32]. For example, EV-mediated signals between tumor cells and stromal cells in the TME, are required in preparing a favorable pre-metastatic niche for disseminated tumor cells. Because EVs are membranous vesicles with a lipid bilayer, vulnerable biomaterials such as RNAs and growth factors are protected from the extracellular environment, allowing efficient interactions between cells that are distant from one another.

This review focuses on our current understanding of how EVs modulate the efficacy of anticancer drugs in the context of TNBC. I use the collective term “EVs” instead of the subgroup terms used in each paper. I analyze published reports on EVs derived from TNBC cells (TNBC-EVs) and their roles in drug resistance and discuss strategies for overcoming the TNBC-EV-mediated resistance.

## 2. Tumor-Derived EVs

**Figure 1:**
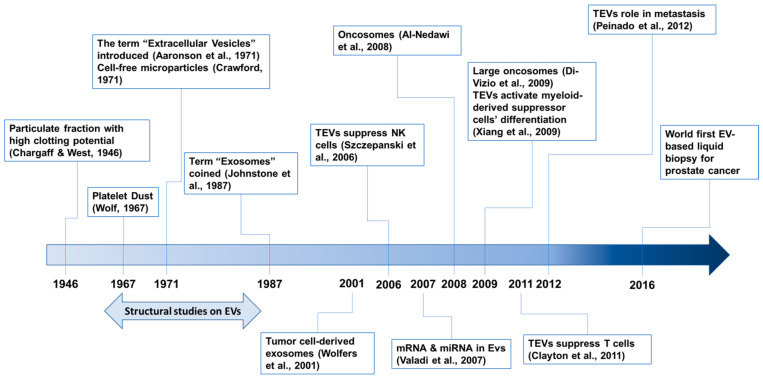
Timeline of selected events in the field of tumor-derived EV (TEV) research. References are cited: Aaronson et al, 1971 [42]; Al-Nedawi et al., 2008 [45]; Chargaff & West, 1946 [39]; Clayton et al., 2011 [46]; Crawford, 1971 [41]; Di-Vizio et al., 2009 [47]; Johnstone et al., 1987 [43]; Peiando et al., 2012 [48]; Szczepanski et al., 2006 [49]; Valadi et al., 2007 [50]; Wolf, 1967 [40]; Wolfers et al., 2001 [51]; Xiang et al., 2009 [52].

The history of EV research has been recently described [33,37,38], although no consensus has been established yet. Our awareness of EVs began in 1946 with a series of papers by Chargaff [39] (Figure 1). The first electron microscopic observations of EVs, described as “platelet dust”, were published in 1967 [40]. In 1971, cell-free microparticles were described [41], and the term “extracellular vesicles” was also introduced [42]. The term “exosomes” was coined in 1987 [43]. EV structural studies were performed during the mid-1960s and early 1980s [37]. Now, EVs are known to play pivotal roles in inter-cellular communications and no longer regarded as gavage disposal tools [34]. In addition, EVs originating from diseased tissues or cells, including cancer cells, are involved in disease progression and recurrence [34,44].

Tumor-derived EVs (TEVs) were first described in 2001 in human and mouse tumor cells [51]. Because TEVs contain and transfer tumor antigens to dendritic cells, they represent a new source for eliciting antitumor immune responses [51]. EVs also transfer functional genetic materials such as mRNA and miRNA between cells [50]. In 2008, the EV transfer of oncogenic proteins between tumor cells was reported [45]. EGFRvIII, an oncogenic mutant of EGFR, is amplified in many glioblastoma multiforme (GBM) patients [53] and is transferred between glioma cells via membranous microvesicles (so-called oncosomes). EGFRvIII activates oncogenic signaling pathways, including mitogen-activated protein kinase (MAPK) and v-akt oncogene homolog (AKT), in recipient cells [45]. TEVs also modulate recipient cells to provide a favorable environment for metastasis [48]. Large oncosomes with diameters up to 10 μm have also been identified [47]. The potential using TEVs for early diagnosis was initially proposed with an in vivo pancreatic cancer model, by detecting the level of TEV-bearing tumor markers [54]. Now, circulating TEVs are generally viewed as diagnostic biomarkers for many types of cancer [55,56], and a diagnostic kit using TEV detection for prostate cancer is commercially available [57,58]. TEVs also play immunosuppressive roles by attenuating the function of natural killer cells [49,59] and T cells [46], and by enhancing the differentiation of myeloid-derived suppressor cells [52].

## 3. TNBC-EVs

Multiple EV cargo components contribute to anticancer drug resistance. Proteins include drug efflux proteins, oncogene products, and signaling pathway components [32].

Two types of non-coding RNAs, microRNAs (miRNAs) and long non-coding RNAs (lncRNAs), are the major RNA components in EVs [32,60,61]. MiRNAs consist of 20–22 nucleotides that bind to the 3′-untranslated region (3′-UTR) of mRNAs and induce degradation or transcriptional inhibition of target mRNAs [62]. LncRNAs contain more than 200 nucleotides and are also involved in transcriptional regulation [63]. The mechanisms for sorting specific RNAs into EVs remain largely unknown [62,64].

### 3.1. TNBC-EV Protein or Nucleic Acid Cargo Components That Induce Anticancer Drug Resistance

Although TNBC tumors express high levels of EGFR [13,22,23,24,25,26], they have intrinsic resistance to EGFR inhibitors (EGFRis) [11,18,19,20,21,65]. Overcoming EGFRi resistance is an alternative therapeutic approach for treating TNBC [11,65]. Although the molecular mechanisms of EGFRi resistance in TNBC are unknown, some have been proposed. For example, compartmentalization of EGFRs within TNBC-EVs could protect them from EGFRis, including erlotinib and gefitinib (reversible), and afatinib (irreversible) [66]. In addition, the transfer of EGFRs in TNBC-EVs can induce signaling pathway activation in recipient TNBC cells, resulting in EGFRi resistance [65].

For TNBC treatment, chemotherapy remains as the main systemic treatment option [67]. Gemcitabine (2′,2′-difluorodeoxycytidine; dFdC) is a preferred treatment option for TNBC patients previously treated with anthracyclines and taxanes [68]. Platinum plus gemcitabine is recommended for metastatic TNBC [69]. However, TNBC rapidly develops resistance to chemotherapy [67]. TNBC resistance to gemcitabine is reportedly transferred via EVs from resistant cells to sensitive cells [68]. Annexin A6 (ANXA6) is elevated in gemcitabine-resistant TNBC cells and their EVs. Mechanistically, ANXA6 interacts with EGFR and blocks its ubiquitination, thereby conferring gemcitabine resistance. The addition of lapatinib, a dual inhibitor for EGFR and HER2 [70], reverses gemcitabine resistance induced by extracellular vesicular ANXA6 (EV-ANXA6). In addition, the levels of ANXA6 in the sera from TNBC patients are predictive of the responsiveness to gemcitabine chemotherapy. As mentioned earlier, the inhibition of EGFR alone is ineffective in TNBC treatment because of intrinsic resistance to EGFRis [11].

Therapeutic-induced senescence (TIS) is involved in the development of drug resistance in cancer cells [71,72]. Senescent Cal51 TNBC cells treated with paclitaxel secrete higher amounts of EVs than non-senescent control cells [73]. In addition, TNBC cells in TIS contain elevated levels of the ATP-binding cassette sub-family B member 1 (ABCB1), and their EVs contain elevated levels of 142 proteins, including ATPases, annexins, tubulins, integrins, RAS-related proteins (RABs), and insoluble senescence-associated secretory phenotype (SASP) factors (fibronectin, collagens alpha-1 and alpha-2, and laminin subunits alpha-5 and beta-1). ABCB1, also known as p-glycoprotein or multidrug resistance protein 1 (MDR1), is an energy-dependent efflux pump that reduces the drug accumulation in drug-resistant cells [74,75,76]. A fluorescent analogue of paclitaxel has been used to show that EV secretion removes the chemotherapy agent from cells in TIS. These findings suggest that cells in TIS maintain their viability, conserving the senescent phenotype, by removing chemotherapy agents and critical proteins involved in cell proliferation, ATP depletion, apoptosis, and the SASP in response to chemotherapeutic challenges [73].

MiRNAs in TNBC-EVs have been reported to promote epithelial-to-mesenchymal transition (EMT) [77], contributing to anticancer drug resistance [78]. Protease-activated receptor 2 (PAR2)-activated TNBC cells produce EVs that induce EMT in recipient cancer cells through the AKT/nuclear factor kappa-light-chain-enhancer of activated B cells (NF-κB) pathway, and contain high levels of miR-221 [77]. MiR-221 downregulates phosphatase and tensin homolog (*PTEN*) mRNA by targeting its 3′-UTR [79]. In addition, miR-221 also targets TNFα-induced protein 3 (TNFAIP3), a ubiquitin-editing enzyme, which inhibits the NF-κB pathway in breast cancer cells [80]. TNBC-EVs containing miR-221 are responsible for inducing cisplatin resistance in sensitive cells [77], and more importantly, EVs derived from TNBC patients also have elevated levels of miR-221, which induces EMT in cancer cells. MiR-221 in EVs derived from colorectal cancer (CRC) cells enhanced liver metastasis by targeting serine protease inhibitor Kunitz type 1 (SPINT1) in recipient cells [81]. In addition, EVs derived from tamoxifen-resistant breast cancer cells transfer tamoxifen resistance to tamoxifen-sensitive ER-positive breast cancer cells in a miR-221-dependent manner [82]. More interestingly, EVs derived from bone marrow mesenchymal stem cells promote the development of acute myeloid leukemia (AML) through miR-221-3p, which directly targets the 3′-UTR of cyclin-dependent kinase inhibitor 1C (*CDKN1C*) that encodes p57KIP2, resulting in the acceleration of the cell cycle, proliferation, and invasion of AML [83].

MiR-423-5p is an EV cargo component whose levels are elevated in EVs derived from cisplatin-resistant TNBC cells compared with EVs derived from cisplatin-sensitive breast cancer cells [84]. EVs derived from cisplatin-resistant TNBC cells transfer cisplatin resistance to sensitive breast cancer cells. Microarray analysis of cisplatin-resistant TNBC-EVs revealed that 60 miRNAs were upregulated and 12 miRNAs were downregulated. A more detailed study of miR-423-5p showed that a miR-423-5p inhibitor suppressed acquired resistance in recipient cells and attenuated the miR-423-5p-induced expression of ABCB1. Interestingly, EVs derived from cancer-associated fibroblasts (CAFs) contain miR-423-5p, and these CAF-EVs promote taxane resistance in prostate cancer by targeting gremlin-2 (GREM2) to activate the transforming growth factor beta (TGFβ) pathway [85].

Circular RNAs (CircRNAs) are covalently closed single-stranded RNA molecules lacking poly-adenylated tails [86]. They are expressed ubiquitously across species and exert their functions as transcriptional regulators, miR sponges, protein templates and protein decoys, scaffolds, and recruiters. Circ_0076611 is synthesized by the back-splicing of vascular endothelial growth factor A (VEGFA) exon 7 in TNBC cell lines and tissue samples from patients [87]. Circ_0076611 induces the expression of proliferation-related genes and proangiogenic cytokines such as MYC proto-oncogene (*MYC*), bromodomain-containing protein 4 (*BRD4*)*,* GATA-binding protein 3 (*GATA3*), *VEGFA*, chemokine (C-C motif) ligand 16 (*CXCL16*), and *CXCL1*. Mechanistically, it enhances the translation rate of target mRNAs by interacting with the translation initiation components eukaryotic translation initiation factor 4B (EIF4B) and EIF4G. Circ_0076611 was detected both in EVs derived from TNBC cell lines and in the sera from TNBC patients. More interestingly, circ_0076611 is positively regulated by lncRNA *MALAT1* (metastasis-associated lung adenocarcinoma transcript 1) by inducing the back-splicing of *VEGFA* exon 7 with ID4 (inhibitor of DNA-binding protein). *MALAT1* has also been reported in EVs derived from CRC cells [88], renal cell carcinoma (RCC) cells [89], and the sera from patients with pancreatic ductal adenocarcinoma [90]. *MALAT1* functions by either promoting binding between the ETS proto-oncogene 1 (ETS1) transcription factor and the promoter region of transcription factor CP2 like 1 (TFCP2L) [89], or by sponging miR-26a/26b to activate fucosylation and the phosphoinositide 3-kinase (PI3K)/AKT pathway through fucosyltransferase 4 (FUT4) [88], leading to invasion and metastasis of RCC or CRC cells. However, the roles of EV-circ_0076611 and EV-*MALAT1* in TNBC progression and drug responses remain to be determined.

The expression profiling of circRNAs shows that circRNA *PSMA1* (circPSAM1) exhibited a higher expression level in MDA-MB-231 cells and EVs than in non-TNBC MCF7 cells and EVs, and in TNBC-EVs derived from TNBC patients and TNBC cell lines compared with those from non-TNBC patients and non-TNBC cells [91]. Overexpression and knockdown experiments further demonstrated that circPSAM1 promotes the proliferation, migration, and cell cycle progression of TNBC cells and inhibits apoptosis in TNBC cells. Mechanistically, circPSAM1 functions as a sponge for miR-637, a negative regulator of AKT, leading to the expression of cyclin D1 and β-catenin, downstream effectors of AKT. EVs carrying circPSAM1 transfer the capacity of cell proliferation and migration to recipient cells via the circPSAM1/miR-637/AKT/β-catenin pathway. However, the role of *PSAM1* in TNBC drug resistance remains to be elucidated.

An analysis of the Cancer Genome Atlas (TCGA) dataset revealed that the lncRNA small ubiquitin-like modifier 1 pseudogene 3 (*SUMO1P3*) is upregulated in breast cancer tissues [92]. Further analysis demonstrated that the expression of *SUMO1P3* in serum EVs was higher in patients with TNBC compared to controls, including patients with non-TNBC and with benign breast disease, and healthy donors. In addition, the levels of serum EV-*SUMO1P3* were highly correlated with lymphovascular invasion, lymph node metastasis, and the histological grade of TNBC. Interestingly, better overall survival (OS) was observed in chemosensitive TNBC patients with lower levels of *SUMO1P3* in serum EVs than in patients with high levels of *SUMO1P3* in serum EVs. In non-small cell lung cancer (NSCLC), *SUMO1P3* is associated with clinical progression, and it promotes cell migration and invasion by binding and suppressing miR-136, an anti-oncogenic miRNA in human cancers [93]. More recently, *SUMO1P3* upregulation has been reported to promote cell proliferation, invasion, and resistance to cisplatin and 5-FU in gastric cancer [94]. *SUMO1P3* directly binds to cellular nucleic acid-binding protein (CNBP), leading to upregulation of CNBP downstream oncogenes, such as MYC and cyclin D1. However, the role of *SUMO1P3* in TNBC-EVs in terms of drug resistance remains to be determined.

TNBC-EV cargo Components that contribute to anticancer drug resistance in TNBC are summarized in Table 1.

### 3.2. Other TNBC-EV Cargo Component That Induces Anticancer Drug Resistance

A recent study demonstrated that TNBC-EVs derived from chemo-resistant cells increase chemoresistance in chemo-sensitive cancer cells by transferring mitochondria with mutations in the *MT-ND4* gene [95]. *MT-ND4* encodes an NADH-ubiquinone oxidoreductase chain 4 protein, one of seven components of the respiratory complex I. A mutated *MT-ND4* gene increased the levels of reactive oxygen species (ROS) in chemo-resistant TNBC cells and those that acquired chemoresistance via TNBC-EVs.

### 3.3. TNBC-EV Cargo Components That Sensitize Drug Response

MiRNAs that are selectively downregulated in drug-resistant TNBC cells and their EVs sensitize TNBC cells to anticancer drugs. EVs derived from an invasive isogenic subclone of the HS578T TNBC cell line (Hs578Ts(i)_8_) [96] were compared with the parental HS578T cell line. MiR-134 was the most substantially suppressed miRNA in Hs578Ts(i)_8_-EVs. MiR-134 suppresses heat shock protein 90 (HSP90) by targeting the 3′-UTR of signal transducer and activator of transcription 5B (*STAT5B*) mRNA [97]. Levels of miR-134 were also lower in tumor tissues of patients with breast cancer. However, the overexpression of miR-134 in Hs578Ts(i)_8_ cells failed to enhance the efficacy of HSP90 inhibitors, such as 17-AAG and PU-H71, but increased the sensitivity of cells to cisplatin. Interestingly, EVs derived from miR-134-transfected Hs578Ts(i)_8_ cells downregulated the levels of STAT5B and HSP90 proteins in Hs578Ts(i)_8_ parental cells and enhanced their sensitivity to HSP90 inhibitors. In contrast to transfection with miR-134, treatment with miR-134-enriched EVs did not affect proliferation or cisplatin sensitivity in Hs578Ts(i)_8_ cells. The reason behind this discrepancy remains unclear. Notably, miR-134 itself has been reported to sensitize cisplatin-resistant human lung adenocarcinoma cells to cisplatin, vincristine, and 5-fluorouracil by targeting the 3′-UTR of Forkhead box M1 (*FOXM1*) mRNA, leading to the downregulation of multidrug resistance-associated protein 1 (MRP1) protein levels [98], and has also been reported to sensitize AML cells to cytarabine by targeting the 3′UTR of MAPK signaling-interacting kinase 1 (*MNK1*) and 2 (*MNK2*) mRNAs [99].

**Figure 2:**
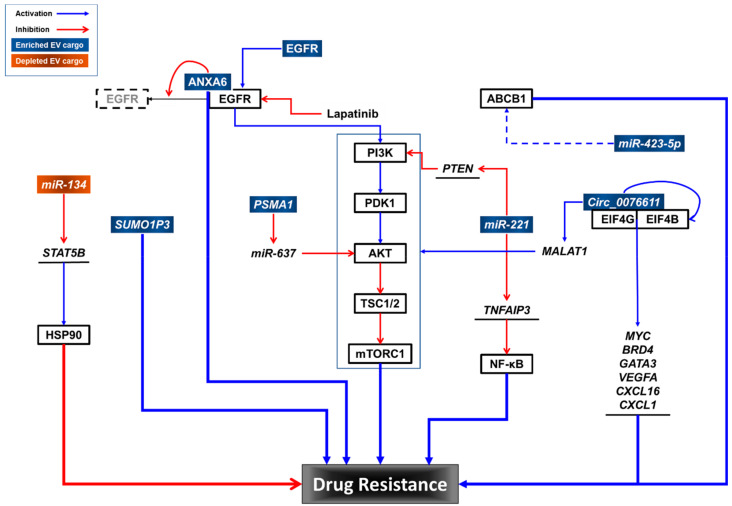
Potential roles of TNBC-EV cargo components in drug resistance.

These data suggest that multiple cargo components of TNBC-EVs may contribute to drug resistance in TNBC cells. Further studies are needed to elucidate the roles of TNBC-EV cargo in drug resistance and to identify potential interventions to overcome drug resistance in TNBC (Figure 2).

### 3.4. TNBC-EV Cargo Components That Modulate the TME and Immune Cells

EVs also mediate crosstalk between cancer cells and the TME. Microenvironment signals regulate macrophage polarization, resulting in M1- or M2-type macrophages. M1 macrophages are inflammatory or anti-tumorigenic, whereas M2 macrophages are anti-inflammatory or pro-tumorigenic [100]. Several studies suggest that TEVs regulate macrophage polarization to support a favorable microenvironment for tumor growth and metastasis [101,102,103,104,105,106]. TNBC-EVs labeled with a red fluorescence protein (RFP) have been utilized to visualize the effect of TNBC-EVs on macrophage polarization [107]. RFP-tagged TNBC-EVs were produced by overexpressing the RFP-fused cluster of differentiation 63 (CD63) in MDA-MB-231 cells. These RFP–TNBC-EVs enhanced cell migration and proliferation in TNBC cells in vitro. They also promoted the migration and M2 polarization of RAW264.7 cells (a macrophage cell line) in vitro, as well as of macrophages residing in axillary lymph nodes (LN) in non-tumor-bearing mice. Intravenous administration of RFP-tagged TNBC-EVs into orthotopic breast cancer-bearing mice promoted tumor metastasis to axillary LNs, with an increased M2/M1 ratio [107]. TNBC-EVs loaded with programmed death-ligand 1 (PD-L1) were found to mediate M2 macrophage polarization by activating the TANK-binding kinase 1 (TBK1)/STAT6 signaling pathway and suppressing the AKT/mammalian target of rapamycin (mTOR) signaling pathway [108].

In another study, MDA-MB-231-derived TNBC-EVs promoted monocyte differentiation toward pro-inflammatory tumor-associated macrophages (TAMs) [109]. TNBC-EVs promote macrophage activation via surface colony stimulating factor 1 (CSF1) and macrophage survival via cargo components that induce the cyclic GMP–AMP synthase (CGAS)/stimulator of interferon genes (STING) pathways. In addition, TNBC-EVs promoted the interferon response in macrophages in vitro through CGAS/STING pathway activation. CSF1-containing EVs were also isolated from human TNBC tissues cultured ex vivo. Macrophages with the TNBC-EV-induced signature were identified in patients’ TAMs, and the expression of this signature is positively associated with the infiltration of T cells and extended patient survival.

Endoplasmic reticulum (ER) stress occurs more frequently in TNBC tissues than in para-cancerous tissues [110]. ER stress is caused when the demand for protein folding exceeds the ER’s capacity [111]. ER stress is favorable for malignant transformation [111] and immune evasion [110]. ER stress induces the infiltration of CD68+ and PD-L1+ macrophages into the tumor stroma. Mechanistically, ER stress enhances the secretion of EVs that contain miR-27a-3p from TNBC cells, and EV-miR-27a-3p upregulates PD-L1 expression in macrophages in vitro and in vivo. MiR-27a-3p targets membrane-associated guanylate kinase inverted 2 (MAGI2), a positive regulator of PTEN, in macrophages. Because inhibition of the PI3K/AKT pathway by PTEN inhibits PD-L1 expression [112,113,114], blocking MAGI2 by miR-27a-3p upregulates PD-L1. A co-culture of macrophages treated with an EV-miR-27a-3p mimic reduced the number of CD8+ T cells in vitro. These data suggest that TNBC-EVs containing miR-27a-3p may positively regulate PD-L1 expression in macrophages to escape immune surveillance by CD8+ T cells. The mechanism by which ER stress upregulates miR-27a-3p in TNBC-EVs by ER stress remains to be elucidated.

The upregulation of PD-L1 by tumor cells increases PD-L1 binding to programmed cell death protein 1 (PD1) on cytotoxic CD8+ T cells, and causes CD8+ T cell dysfunction [115]. PD-L1/2 has been found in TNBC-EVs or EVs isolated from the plasma from patients with TNBC [108,116,117]. PD-L1 in MDA-MB-231-derived EVs suppresses T cell-mediated killing of recipient breast cancer cells [116]. Interestingly, transforming growth factor beta (TGF-β) in the TME enhances PD-L1 loading into EVs [118]. Inhibition of EV-PD-L1 with macitentan (MAC), an FDA-approved drug that targets endothelin receptor A (ETA), increases CD8+ T cell-mediated tumor killing in vivo [119]. MAC inhibits the binding of EV-PD-L1 to PD-1, enhancing the antitumor effects of anti-PDl-1 antibodies by increasing the number of CD8+ T cells and reducing the number of regulator T cells (Tregs). Because exogenous EV-PD-L1 reverses the antitumor effect of MAC, targeting EV-PD-L1 is an alternative approach for treating TNBC and other cancers.

High numbers of PD-L2-bearing EVs, but not PD-L1-bearing EVs, have been reported in EVs obtained from TNBC patient plasma compared to healthy controls [117]. The proportion of EVs was more than tenfold higher in patients’ plasma. Increased numbers of PD-L2-bearing EVs were inversely correlated with OS and the pathological complete response (pCR) to chemotherapy, suggesting that EVs containing PD-L2 could be an early biomarker to identify TNBC patients with a high risk of relapse. However, an association of PD-L2-bearing EVs with drug sensitivity has yet to be demonstrated.

Interestingly, miR-770, a monocyte-derived miRNA, has been reported to suppress doxorubicin resistance in TNBC cells through EV-mediated transfer [120]. MiR-770 inhibits apoptosis, migration, and invasion, and regulates the TME by directly targeting the 3′-UTR of the stathmin gene (*STMN1*). Clinically, miR-770 expression is low in chemo-resistant TNBC tissues, and high expression is associated with better OS in all types of breast cancer. Overexpression of miR-770 in THP-1 human monocytic cells increases the expression of M1 markers, including monocyte chemotactic protein 1 (*MCP1*), inducible NO synthase (*NOS2*), and *CD80*, and decreases the expression of M2 markers, such as *CD206*, arginase-1 (*ARG1*), and macrophage mannose receptor 2 (*MRC2*). When TNBC cells were exposed to the conditioned medium from miR-770-transfected THP-1 cells, the TNBC cells became sensitized to doxorubicin. EVs derived from miR-770 agomir-transfected A549 NSCLC cells inhibit M2 polarization by targeting mitogen-activated protein kinase kinase kinase 1 (MAP3K1) in macrophages [121]. However, direct evidence for the mechanisms of miR-770 in EVs derived from TAMs or chemo-sensitive TNBC cells in TNBC drug resistance remains to be elucidated.

MiRNAs from MDA-MB-231 cells modulate the TME, by specifically stimulating normal fibroblasts to CAFs [122]. MDA-MB-231-EVs induce fibroblast-mediated collagen contraction and fibroblast migration with concomitant increases in the expression of CAF markers, including fibroblast activation protein alpha (*FAP*), caveolin-1 (*CAV1*), solute carrier family 16 member 3 (*SLC16A3*), and *SCL2A1* mRNAs. In addition, MDA-MB-231-EV-activated fibroblasts induce normal breast epithelial MCF10A cells to become invasive in vitro. MiRNA profiling reveals that miR-185-5p, miR-652-5p, and miR-1246 are upregulated in CAFs activated by MDA-MB-213-EVs. These miRNAs each contain short sequence motifs (EXOmotifs) [123] for EV loading. Interestingly, although individual miRNAs have little or no effect on CAF activation, combinations of these miRNAs synergistically activate normal fibroblasts to a pro-migratory CAFs, with the upregulation of integrin A5 and B1 and matrix metalloprotease 1, 2, and 3.

Transmembrane proteins on the surface of TNBC-EVs also contribute to the metabolic reprogramming of CAFs. Upregulation of integrin β (ITGB4) is associated with tumor progression [124]. Interestingly, ITGB4 is not expressed in CAFs, but its expression is induced by contact with TNBC cells [125]. ITGB4 induction in CAFs is mediated by EVs derived from a subset of TNBC cell lines. The TNBC-EV-mediated overexpression of ITGB4 induces glycolysis and mitophagy in CAFs. Mitophagy induction also depends on the induction of BCL2-interacting protein 3-like (BNIP3L), possible via the TNBC-EV-mediated AMPK pathway. In addition, lactate from TNBC-EV-induced glycolysis in CAFs is available for export to TNBC cells as an alternative energy source for cancer progression.

The lipid components of TEVs have not been well explored. However, some studies report that differences in EV lipid compositions depend on parental cell origin [126,127,128]. EVs derived from cell lines with high-metastatic activity have different lipid compositions than those from cell lines with low metastatic activity, even when both cell lines are derived from primary tumors induced by orthotopic xenografts of MDA-MB-231 cells in a mouse [129]. In addition, highly metastatic TNBC cells secrete EVs enriched with unsaturated diacylglycerols (DGs), while causing no increase of DGs in cells. Because DGs in TNBC-EVs activate the protein kinase C and D (PKC and PKD) signaling pathways in HUVECs, but not TNBC cells in vitro, DG-enriched TNBC-EVs could activate endothelial cells to stimulate angiogenesis in the TME.

TNBC-EV cargo components that regulate the TME and immune systems are summarized in Table 2.

### 3.5. Roles of TNBC-EVs in Metastasis

Multiple studies have suggested that TNBC-EVs are initiators of distant metastases and modulators of the premetastatic microenvironment [130,131,132,133] (Table 3). In a mouse model, TNBC tumor-bearing mice exhibit an enhanced expression of extracellular matrix proteins, such as fibronectin, tenascin-C, and periostin, in the lungs compared to non-TNBC tumor-bearing mice [134]. A similar increase in these proteins was also observed in lung fibroblasts treated with TNBC-EVs. However, the molecular mechanisms by which TNBC-EVs modulated these premetastatic microenvironments have not been elucidated.

Comparative proteomic profiling of serum EVs derived from TNBC patients and healthy donors has identified the enrichment of a tetraspanin CD151 in TNBC-EVs [135]. CD151 has been reported to promote tumor metastasis in breast cancer [141,142], hepatocellular carcinoma [143,144], osteosarcoma [145], RCC [146], and clear cell sarcoma [147] in vitro and/or in vivo, by activating signaling pathways, such as PI3K/AKT, rat sarcoma virus protein (RAS)/rapidly accelerated fibrosarcoma (RAF)/extracellular signal-regulated kinase (ERK), or TGFβ. CD151 levels are also more elevated in TNBC cells than in non-TNBC cells [135]. EVs derived from MDA-MB-231 also contain elevated levels of CD151 and induce the migration and invasion of CD151-low TNBC cells in a CD151-dependent manner. Interestingly, CD151 knockout by CRISPR-Cas9 in MDA-MD-231 cells results in altered EV sorting of ribosomal and complement proteins. However, the roles of these alterations by CD151 have not been elucidated yet.

Matrix metalloproteinase 1 (MMP1) is enriched in EVs derived from the MDA-MB-213-HM cell line, a highly pulmonary-metastatic variant of MDA-MB-231 cells [136]. These 231-HM-EVs enhance the migration and invasion activities of various TNBC cells, such as MDA-MB-468 and BT549, as well as the parental cell line. Mechanistically, MMP1-enriched EVs enhance MMP1 secretion from recipient cells, potentially initiating EMT by interacting with its receptor protease activated receptor 1 (PAR1). The 231-HM-EVs promote more tumor metastasis in vivo than 231-EVs. MMP1-shRNA transduction significantly reduces the metastasis-promoting activity of EVs derived from transduced cells. In addition, MMP1 concentrations are higher in EVs from clinical samples of patients with metastasis than those in pre-operative patients, and are higher in patients with multiple distant metastases than in patients with a single lesion.

Nucleoside diphosphate kinases (NDKs) transfer gamma phosphates from ATP to NDP molecules and have been found in EVs derived from various human cancer cell lines [137]. Cell surface NDKs may activate P2Y and P2X receptor signaling, thereby stimulating pro-inflammatory and immunosuppressive responses in the TME [148]. The levels of functional NDK B (NDKB), but not NDKA, are higher in EVs derived from MDA-MB-231 cells than in EVs derived from non-tumorigenic human mammary epithelial (HME) cells [137]. In addition, pharmacologic inhibition of NDKB or P2Y1 receptors reveals that MDA-MB-231-EVs enhance the migration and permeabilization of human umbilical vesicular endothelial cells (HUVEC) in vitro, as well as the lung metastasis of MDA-MB-231 tumors in vivo, in a receptor-dependent manner.

Pentraxin-related protein 3 (PTX3) is an inflammatory protein rapidly produced and secreted by a variety of cells, including mononuclear phagocytes, dendritic cells, fibroblasts, and endothelial cells, in response to primary inflammatory signals [138]. EVs derived from doxorubicin-treated MDA-MB-231 cells are enriched in PTX3 [149]. Chemotherapy treatment may promote tumor metastasis in a subset of TNBC patients who do not achieve pCR [150,151,152,153,154]. Interestingly, doxorubicin treatment enhances doxorubicin-induced tumor metastasis in mouse xenograft models by priming the premetastatic niche in a PTX3-dependent manner, and promotes the secretion of TNBC-EVs enriched in PTX3 [149]. In addition, administration of TNBC-EVs enhances MDA-MB-231 metastasis in vivo, and these pro-metastatic effects are inhibited by blocking either EV secretion or uptake.

Sperm protein associated with the nucleus on the X chromosome B1 (SPANXB1) has been identified as a potential target of a metastasis suppressor endophilin-A1 [139]. Consistently, depletion of SPANXB1 by siRNA reduces TNBC progression via elevated expression of endophilin-A1, with a concomitant reduction of EMT promoters such as RAS-related C3 botulinum toxin substrate 1 (RAC1), α-actin, vinculin, and focal adhesion kinase (FAK). In addition, SPANXB1 is exclusively detected in the circulating EVs of TNBC patients and not in healthy donors or women with benign breast disease. Because the overexpression of SPANXB1 promotes cell migration/invasion of non-metastatic breast cancer cells, EV-mediated transfer of SPANXB1 may contribute to TNBC metastasis.

TNBC-EV-miRNAs are also involved in TNBC metastasis. MiR-4488 is downregulated in EVs derived from TNBC patient sera [140]. EV sorting of miR-4488 is negatively regulated in a mitochondrial calcium uniporter (MCU)-dependent manner in TNBC cells. When overexpressed, miR-4488 suppresses the angiogenesis of HUVECs by directly targeting the 3′-UTR of C-X3-C motif chemokine 1 (*CX3CL1)* mRNA that encodes fractalkine. Clinically, high levels of MCU are correlated with TNBC, as well as the poor survival of patients with breast cancer [140]. EVs derived from MCU-depleted MDA-MB-231 cells suppress metastatic colonization and angiogenesis in the xenograft tumor. MCU may regulate interactions between miRNAs and RNA-binding proteins (RBPs) by calcium-dependent modulation of the components that sort miRNAs into EVs [155,156]. However, further studies are needed to determine the molecular mechanisms by which the MCU regulates miRNA sorting into EVs.

### 3.6. TNBC-EV Cargo as Potential Biomarkers

Various cargo components of TNBC-EVs in circulation and/or tumor tissues could be predictive biomarkers for TNBC treatment outcomes [55,157,158,159,160,161] (Table 4). For example, in the MCF10A isogenic breast cancer progression model, ANXA2 levels in EVs are highly associated with breast cancer cells’ aggressiveness [162]. Analyses of clinical samples revealed that the levels of ANXA2 are higher in EVs derived from the sera of breast cancer patients than in women without cancer. Moreover, serum EV-ANXA2 levels are upregulated in TNBC patients more than in ER+, HER2 patients, or women without cancer. Interestingly, serum EV-ANXA2 levels are more elevated in TNBC patients of African American descent than in Caucasian Americans. Because serum EVs from TNBC patients promoted angiogenesis in an in vivo Matrigel plug assay in an ANXA2-dependent manner, ANXA2-bearing EVs may affect TNBC tumor progression [163]. ANXA2 is involved in plasminogen-plasmin processing, metalloprotease activation, and ECM degradation [164].

EVs containing lncRNA X-inactive specific transcript (*XIST*) are potential liquid biomarkers of TNBC recurrence [165]. Higher levels of *XIST* have been found in tumor tissues and EVs obtained from tumors, as well as the sera of recurrent TNBC patients, than in non-recurrent patients. In addition, high levels of *XIST* in EVs were associated with poorer OS in TNBC patients.

MiR-373 levels in serum EVs are higher in patients with TNBC than in patients with luminal breast cancer and healthy controls [166]. In addition, the levels of EV-miR-373 are also higher in patients with ER-negative or PR-negative breast cancer than in patients with analogous receptor-positive breast cancers. Interestingly, the overexpression of miR-373 downregulates ER protein expression in breast cancer cells by an unknown mechanism. Although the ectopic expression of miR-373 does not affect cell viability, inhibition of camptothecin-induced apoptosis was observed in MCF7 cells with miR-373 overexpression.

## 4. Biomolecular Corona on the Surface of EVs

A standard method for isolating EVs from biological samples has not yet been established [167,168,169]. Currently, differential ultracentrifugation (UC) remains the most common method to purify EVs from various fluids [33,170]. Other approaches, including size exclusion chromatography, ion exchange chromatography, the antibody-capture method, tangential flow filtration, and precipitation, are also applied to isolate EVs from solution [33,169,170]. However, there is no consensus on the methodologies for EV purification [33,170]. The purity and integrity of isolated EVs may be affected by various factors such as the ionic strength of the isolation buffer, mechanical stress during purification, and impurities from buffer components. The quality of isolated EVs should be guaranteed for consistent and reproducible results. Continuous international efforts provide updated guides for the minimal requirements for studies of EVs [171,172,173,174]. In addition, scalable purification methods are needed to produce the good manufacturing practice (GMP)-grade EVs for clinical applications [33,169,175].

In biological fluids, some macromolecules are membrane-active molecules that can be absorbed and form a protein corona on the surface of EVs [176,177,178,179,180]. A protein corona increases the hydrodynamic diameter of an EV [178] and modulates its function and fate. Critical parameters affected by EV coronas include systemic circulation, biodistribution, and bioavailability [181]. As mentioned earlier, the TNBC-EV-mediated differentiation of monocytes into pro-inflammatory TAMs is stimulated by CSF1 on the surface of TNBC-EVs [109].Other proteins, including milk fat globule-EGF factor 8 (MFGE8), galectin-5, and transforming growth factor beta-1 (TGFβ-1), have been reported to bind to or associate with EV surfaces and have specific roles in apoptotic cell clearance in sepsis, vesicle uptake by macrophages, or recipient cell migration, respectively [182,183,184]. Functional NDKs are also found in TNBC-EVs, which may activate P2Y and P2X receptor signaling pathways and stimulate pro-inflammatory and immunosuppressive responses in the TME [137]. Therefore, EVs isolated without their protein corona might not be fully functional [179,180,185]. Using enzyme nomenclature as an example, we may refer to EVs with a biomolecular corona as holo-EVs and those without a corona as apo-EVs. More consideration is needed in developing procedures for isolating EVs that are stringent enough to remove impurities, but gentle enough to retain the biomolecular corona from various biological fluids.

## 5. Conclusions

As mentioned, studying the roles of EVs in drug resistance still remains in the early stages. However, growing evidence suggests that TEVs and EVs from the TME contribute to the occurrence of drug resistance in tumor cells by transferring various cargo components between cells. TNBC is an intractable malignancy with intrinsic and rapidly developed drug resistance. As many TNBC-EV cargo components have been reported to regulate drug resistance and the TME, more considerations on the mechanism of EV-mediated drug resistance are required to circumvent drug resistance.

Targeting EV components could be an alternative approach for treating cancer. For example, blocking miRNAs such as miR-221 [76] and miR-423-5p [83] is a potential target to overcome cisplatin resistance in TNBC. Targeting PD-L1 [107] or PD-L2 [116] on the surface of TNBC-EVs is also a plausible strategy to induce an immune response against tumors. For example, the FDA-approved drug, MAC, inhibits EV-PD-L1, leading to increased tumor cell killing by CD8+ T cells [118].

Perturbing EV formation or secretion is also another potential target [186]. Pharmacological small molecule inhibitors, including GW4869 or Y27632, are now being explored as research tools and potential therapeutics. Selective delivery of these agents or development of cancer-selective inhibitors remains a challenge for EV inhibitors as anticancer drugs.

In summary, EVs play a key role in drug resistance of TNBC, and pharmacological interventions of EV function are potential therapeutic approaches to treat TNBC.

## Figures and Tables

**Table 1:** TNBC-EV cargo components that confer resistance to anticancer therapeutics.

Cargo	Molecular Type	Feature and Function
ANXA6 (Annexin A6)	Protein	- Upregulated in gemcitabine-resistant TNBC cells and their EVs, and transferred via EVs to gemcitabine-sensitive TNBC cells, leading to gemcitabine resistance in sensitive cells [68]
EGFR (Epidermal growth factor receptor)	Protein	- Sequestered in EVs to protect from EGFR inhibitors and triggers EGFR signaling in recipient cells, leading to increased proliferation and migration. [66]
Insoluble SASP proteins	Protein	- Secreted in EVs from TIS TNBC cells [73].
miR-221	miRNA	- Induces EMT via activation of the AKT/NF-κB pathway and cisplatin resistance in sensitive cancer cells [77]
miR-423-5p	miRNA	- Elevated in EVs from cisplatin-resistant TNBC cells and transfers cisplatin resistance to sensitive cells [84]
Circ_0076611	circRNA	- Induces the expression of its target mRNAs, including MYC and VEGFA mRNAs by enhancing their interaction with translation initiation machinery [87]
CircPSMA1	circRNA	- Promotes cell proliferation, migration, and cell cycle progression of TNBC cells by functioning as a miR-637 sponge to inhibit AKT expression [91]
*SUMO1P3* (Small ubiquitin-like modifier 1 pseudogene 3)	lncRNA	- Upregulated in EVs in the sera of TNBC patients and inversely associated with chemosensitivity and correlated with poor OS [92]

**Table 2:** TNBC-EV cargo components that modulate the TME and immune cells.

Cargo	Molecular Type	EV Source	Feature and Function
CSF1 (Colony stimulating factor 1)	protein	MDA-MB-231 Patient tissues	- Activates monocytes to TAMs by promoting survival and affecting responses to interferon through activation of the cGAS/STING pathway [109]
ITGB4 (Integrin β4)	protein	MDA-MB-231 BT-20	- Induces glycolysis and mitophagy in CAFs [125]
PD-L1	protein	Patient tissues TNBC cells	- Induces macrophage polarization into the M2 phenotype [108] - Highly expressed in tissues derived from TNBC patients [108] - Inhibits the function of CD8+ T cells by interacting with PD1 [108,116,118]
PD-L2	Protein	Patient plasma	- A negative biomarker for risk assessment of TNBC patients with chemotherapy [117]
miR-185-5p miR-652-5p miR-1246	miRNA	MDA-MB-231	- Synergistically activates normal fibroblasts to CAFs in vitro [122] - Upregulates FAP, monocarboxylate transporter 4 (MCT4), CAV1, ITGA5, ITGB1, matrix metalloprotenase-1 (MMP1), MMP2, and MMP3 in CAFs [122]
miR27a-3p	miRNA	Patient tissues	- Upregulates PD-L1 in macrophages in TNBC tumor stroma to facilitate the immune escape of tumor cells [110]
miR-770	miRNA	TNBC cells Patient tissue	- Inhibits apoptosis, migration, and invasion [119] - Regulates the TME by targeting *STMN1* [119] - Polarizes monocytes to M1 macrophages [119]
DGs	Lipid	TNBC cells	- Activates endothelial cells to stimulate angiogenesis in the TME [128]

**Table 3:** TNBC-EV cargo involved in tumor metastasis.

Name	Cargo Type	EV Source	Feature and Function
CD151	Protein	MDA-MB-231 Patient serum	- Enhances migration and invasion of TNBC cells in vitro [135]
MMP1	Protein	MDA-MB-231	- Enhances metastasis of tumors in vivo [136] - Enriched in EVs derived from serum of patients with metastatic TNBC [136]
NDKB	Protein	MDA-MB-231	- Enhances migration and permeabilization of HUVEC in vitro and metastasis of MDA-MB-231 in vivo via NDKB enzyme activity- or a P2Y1 receptor-dependent manner [137]
PTX3	Protein	Doxorubicin-treated MDA-MD-231	- Enhances metastasis of tumors in vivo [138]
SPANXB1	Protein	MDA-MB-231 SUM149 Patient serum	- Promotes cell migration and invasion of non-metastatic breast cancer cells [139]
miR-4488	miRNA	MDA-MB-231 Patient serum	- Downregulated in EVs derived from TNBC cells and serum from TNBC patients in a MCU-dependent manner [140] - Negatively regulates angiogenesis of HUVECs by targeting CX3CL1 [140]

**Table 4:** TNBC-EV cargo components as biomarkers.

Name	Cargo Type	EV Source	Feature and Function
ANXA2	Protein	Patient serum	- Highly expressed in EVs obtained from sera of TNBC patients of African American heritage [163]
XIST (X-inactive specific transcript)	lncRNA	Patient serum	- A negative biomarker for recurrent TNBC-loading status [165]
miR-373	miRNA	Patient serum	- Levels are higher in TNBC patients than in patients with luminal breast cancer or healthy controls [166] - Ectopic expression inhibits camptothecin-induced apoptosis in luminal breast cancer MCF7 cells [166]

## Data Availability

Not applicable.
